# HOME-BIO (sHOtgun MEtagenomic analysis of BIOlogical entities): a specific and comprehensive pipeline for metagenomic shotgun sequencing data analysis

**DOI:** 10.1186/s12859-021-04004-y

**Published:** 2021-07-05

**Authors:** Carlo Ferravante, Domenico Memoli, Domenico Palumbo, Paolo Ciaramella, Antonio Di Loria, Ylenia D’Agostino, Giovanni Nassa, Francesca Rizzo, Roberta Tarallo, Alessandro Weisz, Giorgio Giurato

**Affiliations:** 1 Laboratory of Molecular Medicine and Genomics, Department of Medicine, Surgery and Dentistry ‘Scuola Medica Salernitana’, University of Salerno, Via S. Allende, 1, 84081 Baronissi, SA Italy; 2 Department of Veterinary Medicine and Animal Production, University of Naples Federico II, Via Delpino 1, 80137 Naples, Italy; 3 Genomix4Life, via S. Allende 43/L, 84081 Baronissi, SA Italy; 4 CRGS - Genome Research Center for Health, University of Salerno Campus of Medicine, 84081 Baronissi, SA Italy

**Keywords:** Shotgun metagenomics, Next-Generation-Sequencing, Pipeline

## Abstract

**Background:**

Next-Generation-Sequencing (NGS) enables detection of microorganisms present in biological and other matrices of various origin and nature, allowing not only the identification of known phyla and strains but also the discovery of novel ones. The large amount of metagenomic shotgun data produced by NGS require comprehensive and user-friendly pipelines for data analysis, that speed up the bioinformatics steps, relieving the users from the need to manually perform complex and time-consuming tasks.

**Results:**

We describe here HOME-BIO (sHOtgun MEtagenomic analysis of BIOlogical entities), an exhaustive pipeline for metagenomics data analysis, comprising three independent analytical modules designed for an inclusive analysis of large NGS datasets.

**Conclusions:**

HOME-BIO is a powerful and easy-to-use tool that can be run also by users with limited computational expertise. It allows in-depth analyses by removing low-complexity/ problematic reads, integrating the analytical steps that lead to a comprehensive taxonomy profile of each sample by querying different source databases, and it is customizable according to specific users’ needs.

## Background

Metagenomics is an interdisciplinary research field combining molecular genetics, microbial ecology, and data processing [1]. Nowadays, the advances in high-throughput sequencing technology allow analyzing the total genomic content of clinical or environmental samples, detecting a large number of organisms and viruses at once and allowing to correlate the taxonomic composition to the metabolic processes where they are involved [2, 3]. The main sequencing methods applied for the analysis of metagenome are, the so-called, ‘marker gene’ and ‘shotgun’ sequencing approaches. These are now widely used respect to the standard methods requiring isolation and cultivation of each single microbial species of interest, which can only provide a limited view, even considering that several microorganisms are not easily cultured in vitro [4]. Marker gene approach (e.g., amplicon sequencing of 16S rRNA gene of bacteria), focuses on a polymorphic region of the microbial genome, that is amplified and sequenced in order to infer at once the composition of the (nearly) entire microbial community present in a given sample [5]. This strategy foresees sequencing of PCR-amplified sequences corresponding to the 16S bacterial rRNA gene which shows some distinctive characteristics that make it suitable for taxonomic profiling and analysis in a single step of a large spectrum of bacteria [6]. In contrast, metagenomic shotgun sequencing (e.g. Whole Metagenome, WMG sequencing) targets all microbial nucleic acids isolated from a sample, followed by deep sequencing of the DNAs drawn randomly from the mixture. The main advantage of this procedure is represented by a large amount of data and information obtained. Contrary to 16S marker gene sequencing, this approach avoids PCR amplification, a major source of potential bias, and provides in-depth knowledge of the microbiome present in the sample, consisting not only of bacterial but also of fungal, protozoal, and viral entities [7, 8]. The presence of several microorganisms and their interaction with each other and the host, play an important role in many physio-pathological processes such as reproduction, immune system activity, cancer, and metabolic disorders [9–12]. Data produced by WMG shotgun sequencing consist of large sets of sequenced reads and represents a challenge for bioinformatics analysis and biological interpretation, particularly in clinical samples. WMG data analysis requires the implementation of multiple ad hoc tools designed, whose installation and configuration, including reference library and computational environment variables, is complex and troublesome in particular for investigators with limited computational expertise. Furthermore, most pipelines available for non-expert users are designed to measure the diversity of microbiota composition from environmental samples (e.g. soil, water) rather than their detection and characterization in biological matrices. Moreover, these pipelines do not provide a global view of sample metagenomic content, since generally they have been designed for specific entities [13–16]. In this context, Kodoja [17] is a workflow specifically developed for plant datasets, identifying only viral sequences from mixed RNAseq data. MetaFlow [18], instead, perform taxonomy profiling using genomic sequences (DNA). It is implemented in C++ and needs a specific input file format (LEMON’s LGF): a graph-based representation of the alignments of metagenomic reads in a collection of reference genomes derived from a bacterial domain. Other pipelines such as nf-rnaSeqMetagen [19] are designed for RNAseq data, and therefore of limited use for metagenomics. Furthermore, this pipeline is implemented in Nextflow [20] and all the applications needed to execute the workflow are containerized in Singularity [21]. Nonetheless, it performs quality check step, host filtering, taxonomic profiling of unmapped reads, and, in order to make taxonomic classification faster, it also runs an assembly de novo step with Trinity [22]. Metaphlan2 [23] is another tool for profiling the composition of microbial communities but, in its present version, is unable to query protozoal databases. In addition, other freely available solutions, such as ASaiM [24] or Galaxy [25], are able to perform numerous steps in a metagenomic investigation, incorporating several exhaustive integrated tools. ASaiM, for example, is an Open-Source Galaxy-based framework dedicated to microbiota data analyses, distributed also via Docker and Conda. MGnify [26], instead, provides a platform for the assembly, analysis and archiving of microbiome data, it requires an internet connection and registration. A summary of common and specific features of several freely available metagenomic pipelines is reported in Additional file 1. Therefore, an easy to use, comprehensive and specific analysis workflow for shotgun sequencing from samples with variable content of host genetic material is still unavailable and much required.

Here we describe HOME-BIO (sHOtgun MEtagenomic annotation of BIOlogical entities), a dockerized solution for the analysis of WMG shotgun datasets that aims at addressing and solving the above mentioned limitations and problems. HOME-BIO is a modular and flexible pipeline that allows taxonomic profiling by allowing the operator to choose between two analytical approaches commonly used in WMG shotgun analysis. The first, defined Metagenomic shotgun module, performs taxonomic characterization and abundance estimation based on exact k-mer matches, to achieve high accuracy and fast classification speeds. The Assembly de novo module, instead, provides the assembly of sequences in contigs in order to perform an unbiased analysis of entire genomes sequenced in the sample. Furthermore, the performance of species classification in metagenomics applications can be improved using long reads, assembled from short reads [27]. The two main modules (Metagenomic shotgun and Assembly de novo module) are preceded by a quality control step, which combines quality check of the sequence reads, followed by filtering host and contaminant reads which may interfere with the results. Compared to the other pipelines mentioned above, HOME-BIO shows some common and specific features (Additional file 1). One of the features of HOME-BIO is the additional protein validation step, available in both modules, that makes more robust the taxonomic profiling analysis. Using protein-level classification it is possible to increase the study accuracy [28]. This characteristic makes HOME-BIO exhaustive and accurate, resulting in a flexible and ready-to-use tool in the hands of investigators interested in metagenomics. Indeed, HOME-BIO is platform-independent and does not require time-consuming and tedious installation or dependency issues due to the Docker implementation. Its modularity makes it flexible to users’ needs. In this context, users can choose some input options (DNA or RNA, single or paired-end read sequencing protocols) and query various reference databases in order to obtain comprehensive taxonomic profiling of the metagenomic samples. We tested HOME-BIO on a public dataset deposited in the NCBI database under BioProject/BioSamples with accession number SRP040611 [29]. In their study, Mitra et al. collected atherosclerotic tissue samples from a group of 7 patients that underwent elective carotid endarterectomy following repeated transient ischemic attacks, or minor strokes, and asymptomatic atherosclerotic plaques from 5 controls. Outcomes are reported in a graphical that summarize all experiments analyzed and achieve a better comprehension of the biological entities identified in each sample.

## Implementation

**Fig. 1:**
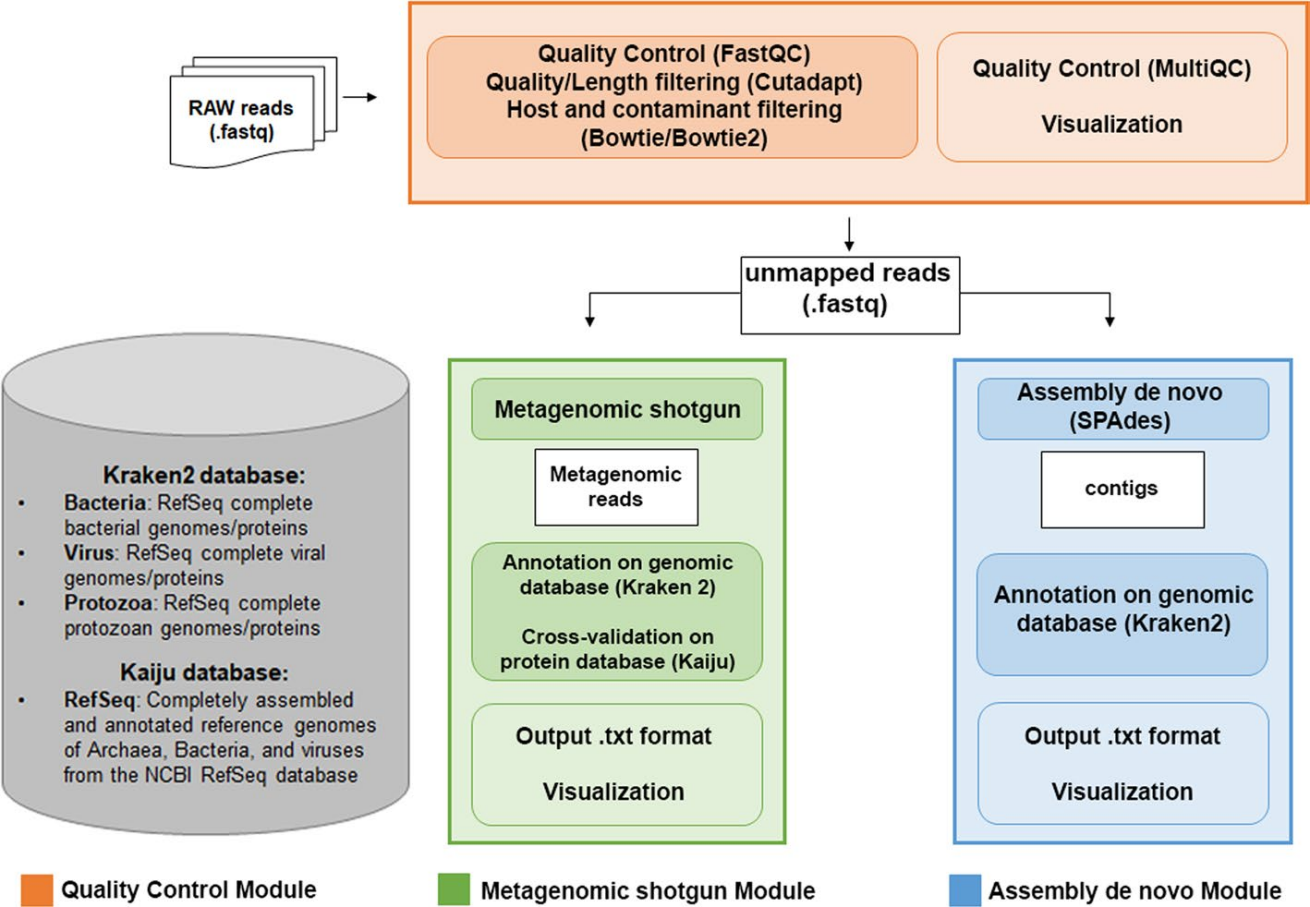
HOME-BIO workflow. The pipeline accepts NGS data as input and then proceeds to perform the analysis according to the different steps comprised in the three modules. The Quality Control module (in orange) performs QCs of the reads, low-complexity reads removal, and host and contaminant sequencing reads filtering (indicated in the dark orange rectangle), generating as output quality reports and graphical charts (indicated in the light orange rectangle). The sequence reads maintained after this step are processed using the Metagenomic shotgun module (in green) and/or the Assembly de novo module (in blue). In the first case, the reads are processed by Kraken2 and Kaiju (indicated in the dark green rectangle), producing as output text tables and charts. The Assembly de novo module processes the sequences by assembling with SPAdes and annotating with Kraken2 (dark blue rectangle), generating as output text tables and charts. For both modules, a custom database (indicates in the grey box) is used

HOME-BIO is implemented using both tools mainly used in metagenomic analyses and custom python scripts to produce tables and charts for immediate and easier interpretation of the results. The central core of this pipeline is its modularity, being composed of three main blocks: ‘Quality Control’, ‘Metagenomic Shotgun’ and ‘Assembly de novo’ modules (Fig. 1). They can be run all together or separately, according to the user’s needs. The “Quality Control” module allows to perform sequence read quality checks and includes FASTQC [30] and MultiQC [31], to perform the quality check and the summary of quality control, respectively, while the adapter trimming and removal of low-quality reads is performed by Cutadapt [32]. If required, HOME-BIO performs a filtering step to remove host and contaminant sequence fragments by mapping each of them on the corresponding genomes. This alignment is performed with bowtie [33], for input reads with length less than 50 bp and with bowtie2 [34] for those longer than 50 bp. The ‘Metagenomic Shotgun’ module performs taxonomic profiling by classifying unmapped reads with Kraken2 [35]. By default, the confidence score threshold of 0.5 is used to define the quality of taxonomic classification. To extract as much information as possible, bacterial, viral, and protozoal NCBI databases built by Kraken2 are provided. Nonetheless, if users intend to explore only one of the domains described before (or only viral entities), it is possible to set custom options to query only one (or more) databases and obtain the taxonomic information desired. Compared to other taxonomic profiling pipelines freely available, such as MicroPro [36], METAwrap [37], and Sunbeam [38], HOME-BIO performs an additional protein-validation step for non-eukaryotic entities. This implementation makes taxonomic classification outcomes more robust by using protein-level classification with Kaiju [28], thereby increasing the reliability and sensitivity of the analysis. Kaiju carries out a comparison to a reference database containing microbial and viral protein sequences. Unmapped metagenomic input reads are thus translated into amino acid sequences and then searched in the database using a modified backward search on a memory-efficient implementation of the Burrows-Wheeler transform, which finds maximum exact matches. As default, we set the evaluation in run mode *greedy*, with an e-value cutoff of 0.001. All the entities classified with Kaiju are then processed with Krona [39], to obtain a comprehensive graphic visualization. As outputs, the ‘Metagenomic shotgun’ module generates a table containing the Kranken2 taxonomy profile and related Kaiju protein-validation information. A given taxon is considered protein-validated when both tools classify and assign reads to it. In addition, HOME-BIO generates output pie-charts with the top 15 represented species, with an estimation of the relative abundance of each of them. In the ‘Assembly de novo*’* module analysis, HOME-BIO uses SPAdes [40] in metagenomic mode (option *-meta*). It takes in input unmapped reads resulting from the ‘Quality Control’ module, generating sequence contigs that are classified with Kraken2, and protein-validated with Kaiju as described before. As mentioned above, HOME-BIO takes advantage of several reference databases. This allows users to investigate in depth the content of their biological samples. We linked NCBI taxonomy information from complete RefSeq bacterial, viral, and protozoal genomes/proteins. If it ran in end-to-end mode, the pipeline provides comprehensive profiling of specimens by querying the above-mentioned databases. This aspect makes HOME-BIO a powerful tool in the hands of users dealing with metagenomic data.

To make its installation easier, HOME-BIO is inserted in a Docker [41] image, with installed Ubuntu 18.04.4 LTS and Anaconda 3 (V. 02/2020) (https://anaconda.com). This allows that all the required tools are always compatible with each other, automatically updated, and handled by Anaconda itself. All the parameters are set using a configuration file that is given as input file when the pipeline is launched.

## Results

HOME-BIO processes NGS data to characterize the composition of host-associated microbial communities. The pipeline is implemented to be used by biologists with limited computational experience who intend to explore the pathogens’ content in their samples.

**Fig. 2:**
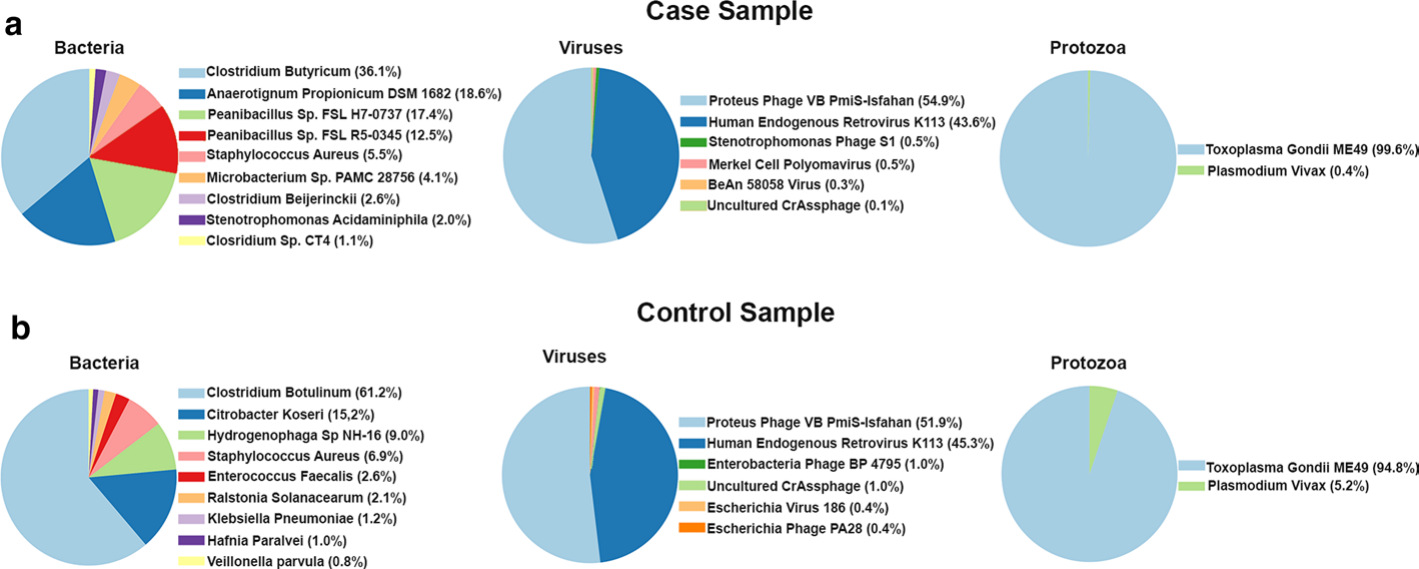
Pie Charts regarding Bacteria, Viruses, and Protozoa detected in the samples. Pie-charts summarizing the percentage of Bacteria, Viruses, and Protozoa detected in samples SRR1206003 (Case) and SRR1202227 (Control) obtained by data analysis with the HOME-BIO pipeline

To test the performance of HOME-BIO, we considered the public dataset of Mitra et al. [29], comprising 12 samples: 7 from symptomatic atherosclerotic plaques and 5 matched controls from asymptomatic atherosclerotic plaques. The analysis was performed running all three modules with default parameters and using the databases provided with the pipeline [42], comprising complete RefSeq bacterial, viral, and protozoa genome sequences and a subset of NCBI BLAST non-redundant database containing all proteins belonging to Archaea, Bacteria, and Viruses. To extract more confident sequences to be assigned to biological entities, the reads were mapped against the genome of the host (human genome assembly hg19). After running the Quality Control module, the number of unmapped reads, usable in the next computational step, ranged from 6.449.408 to 14.710.470, with an average value of 7.991.03720 for Controls and 11.841.55800 for Cases samples, representing 26.17% and 41.63% of the total reads in Control and Case, respectively. Subsequently, for each sample, the reads were analyzed with the Shotgun Metagenomic module, in order to obtain the taxonomy profile. HOME-BIO queried bacterial, viral, and protozoal databases. The Quality Control and Metagenomic results are summarized in Table 1 and Fig. 2 and are reported in Additional file 2: Tables S1–S24, Additional file 3: Tables S1–S24, and Additional file 4: Tables S1–S24.

**Table 1:** Total number and percentage of reads assigned to Bacteria, Viruses, and Protozoa for each analyzed sample

Sample-name	Raw reads	Non-hg19%	HOME-BIO shotgun Bacteria reads classified (%)	HOME-BIO shotgun Virus reads classified (%)	HOME-BIO shotgun Protozoa reads classified (%)
SRR1205226	62.241.508	10.36	4617 (0.07)	331 (0.01%)	177713 (2.76)
SRR1205227	83.273.796	8.79	259578 (3.54)	595 (0.01%)	237811 (3.25)
SRR1205228	57.336.562	16.43	5955 (0.06)	677 (0.01%)	218329 (2.32)
SRR1205230	75.597.642	11.50	52529 (0.6)	2496 (0.03)	262034 (3.01)
SRR1205231	76.089.590	10.60	5316 (0.07)	697 (0.01)	217939 (2.7)
SRR1205232	70.667.046	20.82	7420 (0.05)	1152 (0.01)	278959 (1.9)
SRR1206003	76.651.484	12.06	139334 (1.51)	736 (0.01)	264054 (2.86)
SRR1206005	77.326.222	15.57	8235 (0.07)	720 (0.01%)	264974 (2.2)
SRR1206007	50.795.748	22.63	3713 (0.03)	684 (0.01)	174687 (1.52)
SRR1206009	49.963.912	21.03	4393 (0.04)	491 (0.00)	202508 (1.93)
SRR1206011	41.425.332	28.19	3434 (0.03)	732 (0.01%)	196978 (1.69)
SRR1206012	52.047.446	25.38	5146 (0.04)	748 (0.01)	230780 (1.75)

Running the Assembly de novo module, 567.37957 contigs for Cases samples and 238.7888 contigs for Control samples were obtained. The results are summarized in the Additional file 5: Tables S1–S24, Additional file 6: Tables S1–S24, and Additional file 7: Tables S1–S24. We identified an average of 5.5752 and 12.9563 contigs classified as bacteria in Control and Cases samples, respectively. Among these, 4554 in Control and 816 in Cases were contigs classified as viruses, while 12.2198 contigs in Control and 18.4238 in Cases matched in the protozoal database. These results obtained on bacteria from the analysis of the 12 samples are in agreement with the ones described by Mitra et al. [29]. In addition, HOME-BIO allowed to identify also several other viruses and protozoa that could be related to the disease and are reported in Additional file 3: Tables S1–S24, Additional file 4: Tables S1–S24 comprised the reports from the Metagenomics module, and Additional file 6: Tables S1–S24 and Additional file 7: Tables S1–S24 those from the Assembly de novo module.

For a further test with a different species, HOME-BIO was used to analyze a small dataset derived in our lab by sequencing four samples from canine heart biopsies, two consisting of formalin-fixed, paraffin-embedded (FFPE) samples and two of snap-frozen biopsies (raw data available on Array Express with Accession n.er E-MTAB-9189 and E-MTAB-9191). Results confirmed the good performance of the tool in all these datasets, leading as expected to the identification of several microorganisms independent from the nature and pre-treatment of the tissue samples. The results from all these samples are included in Additional file 8: Tables S1–S2. A tutorial with figures and examples of usage is included in Additional file 9.

## Conclusions

Here, we describe HOME-BIO, a user-friendly pipeline based on a dockerized solution, designed for analyzing shotgun metagenomic data avoiding time-consuming and error-prone installation and configuration steps. This modular pipeline provides a quality control step and the two main analysis approaches commonly used in metagenomic studies. By querying bacterial, viral, protozoal, and protein databases, HOME-BIO generates also an exhaustive taxonomic profiling of the biological entities in specimens.

### Availability and requirements

- Project name: HOME-BIO
- Project home page: https://github.com/carlferr/HOME-BIO
- Operating system(s): Any
- Programming language: Python
- Other requirements: Docker
- License: GNU GPL
- Any restrictions to use by non-academics: none

## Supplementary Information

**Additional file 1: Table S1**. Characteristics of Shotgun Pipelines.

**Additional file 2: Table S1–S24**. HOME-BIO reports for bacteria identified with the metagenomic module in the twelve samples analyzed.

**Additional file 3: Table S1–S24**. HOME-BIO reports for viruses identified with the metagenomic module in the twelve samples analyzed.

**Additional file 4: Table S1–S24**. HOME-BIO reports for protozoa identified with the metagenomic module in the twelve samples analyzed.

**Additional file 5: Table S1–S24**. HOME-BIO reports for bacteria identified with the Assembly de novo module in the twelve samples analyzed.

**Additional file 6: Table S1–S24**. HOME-BIO reports for viruses identified with the Assembly de novo module in the twelve samples analyzed.

**Additional file 7: Table S1–S24**. HOME-BIO reports for protozoa identified with the Assembly de novo module in the twelve samples analyzed.

**Additional file 8: Table S1–S2**. HOME-BIO reports for bacteria, viruses, and protozoa identified Can_Fam samples.

**Additional file 9**. Tutorial and use examples of HOME-BIO.

## Data Availability

The data used in this study are available on NCBI with accession number SRP040611 and on ArrayExpress with accession number E-MTAB-9189 and E-MTAB-9191. Pre-indexed Kaiju and Kraken2 databases are available on Zenodo [42] (https://doi.org/10.5281/zenodo.4055180) and a test dataset is available on Zenodo [43] (https://doi.org/10.5281/zenodo.4061297).

## Abbreviations

NGS: Next-Generation Sequencing
WMG: Whole Metagenome

## References

[1] Sudarikov K, Tyakht A, Alexeev D. Methods for the metagenomic data visualization and analysis. Vol. 24, Current issues in molecular biology. Curr Issues Mol Biol; 2017. p. 37–58. 10.21775/cimb.024.037 28686567

[2] Mendes LW, Braga LPP, Navarrete AA, de Souza DG, Silva GGZ, Tsai SM (2017). Using metagenomics to connect microbial community biodiversity and functions. Curr Issues Mol Biol.

[3] Quince C, Walker AW, Simpson JT, Loman NJ, Segata N. Shotgun metagenomics, from sampling to analysis. Vol. 35, Nature Biotechnology. Nature Publishing Group, Berlin; 2017. p. 833–44. 10.1038/nbt.3935 28898207

[4] Lagier JC, Dubourg G, Million M, Cadoret F, Bilen M, Fenollar F, et al. Culturing the human microbiota and culturomics. Vol. 16, Nature Reviews Microbiology. Nature Publishing Group, Berlin; 2018. p. 540–50. 10.1038/s41579-018-0041-0 29937540

[5] Turnbaugh PJ, Ley RE, Hamady M, Fraser-Liggett CM, Knight R, Gordon JI (2007). The human microbiome project.

[6] Amit Roy SR (2014). Molecular markers in phylogenetic studies—a review. J Phylogenetics Evol Biol.

[7] Marotz CA, Sanders JG, Zuniga C, Zaramela LS, Knight R, Zengler K (2018). Improving saliva shotgun metagenomics by chemical host DNA depletion. Microbiome.

[8] Conrads G, Abdelbary MMH (2019). Challenges of next-generation sequencing targeting anaerobes.

[9] Benson AK, Kelly SA, Legge R, Ma F, Low SJ, Kim J (2010). Individuality in gut microbiota composition is a complex polygenic trait shaped by multiple environmental and host genetic factors. Proc Natl Acad Sci USA.

[10] Atreya CE, Turnbaugh PJ (2020). Probing the tumor micro(b)environment. Science (80-).

[11] Nejman D, Livyatan I, Fuks G, Gavert N, Zwang Y, Geller LT (2020). The human tumor microbiome is composed of tumor type–specific intracellular bacteria. Science (80-).

[12] Zitvogel L, Ma Y, Raoult D, Kroemer G, Gajewski TF (2018). The microbiome in cancer immunotherapy: diagnostic tools and therapeutic strategies.

[13] Ji Y, Huotari T, Roslin T, Schmidt NM, Wang J, Yu DW (2020). SPIKEPIPE: a metagenomic pipeline for the accurate quantification of eukaryotic species occurrences and intraspecific abundance change using DNA barcodes or mitogenomes. Mol Ecol Resour.

[14] Milani C, Casey E, Lugli GA, Moore R, Kaczorowska J, Feehily C (2018). Tracing mother-infant transmission of bacteriophages by means of a novel analytical tool for shotgun metagenomic datasets: METAnnotatorX. Microbiome.

[15] Piper AM, Batovska J, Cogan NOI, Weiss J, Cunningham JP, Rodoni BC (2019). Prospects and challenges of implementing DNA metabarcoding for high-throughput insect surveillance. Gigascience.

[16] Rampelli S, Soverini M, Turroni S, Quercia S, Biagi E, Brigidi P (2016). ViromeScan: a new tool for metagenomic viral community profiling. BMC Genomics.

[17] Baizan-Edge A, Cock P, MacFarlane S, McGavin W, Torrance L, Jones S (2019). Kodoja: A workflow for virus detection in plants using k-mer analysis of RNA-sequencing data. J Gen Virol.

[18] Sobih A, Tomescu AI, Mäkinen V (2016). Metaflow: Metagenomic profiling based on whole-genome coverage analysis with min-cost flows. Lect Notes Comput Sci (including Subser Lect Notes Artif Intell Lect Notes Bioinformatics).

[19] Mpangase PT, Frost J, Ramsay M, Hazelhurst S (2020). nf-rnaSeqMetagen: a nextflow metagenomics pipeline for identifying and characterizing microbial sequences from RNA-seq data. Med Microecol.

[20] Di Tommaso P, Chatzou M, Floden EW, Barja PP, Palumbo E, Notredame C (2017). Nextflow enables reproducible computational workflows. Nat Biotechnol.

[21] Kurtzer GM, Sochat V, Bauer MW (2017). Singularity: Scientific containers for mobility of compute. PLoS ONE.

[22] Grabherr MG, Haas BJ, Yassour M, Levin JZ, Thompson DA, Amit I (2011). Full-length transcriptome assembly from RNA-Seq data without a reference genome. Nat Biotechnol.

[23] Segata N, Waldron L, Ballarini A, Narasimhan V, Jousson O, Huttenhower C (2012). Metagenomic microbial community profiling using unique clade-specific marker genes. Nat Methods.

[24] Batut B, Gravouil K, Defois C, Hiltemann S, Brugère J-F, Peyretaillade E (2018). ASaiM: a Galaxy-based framework to analyze microbiota data. Gigascience.

[25] Afgan E, Baker D, Batut B, van den Beek M, Bouvier D, Čech M (2018). The Galaxy platform for accessible, reproducible and collaborative biomedical analyses: 2018 update. Nucleic Acids Res.

[26] Mitchell AL, Almeida A, Beracochea M, Boland M, Burgin J, Cochrane G (2019). MGnify: the microbiome analysis resource in 2020. Nucleic Acids Res.

[27] Tran Q, Phan V (2020). Assembling reads improves taxonomic classification of species. Genes (Basel).

[28] Menzel P, Ng KL, Krogh A (2016). Fast and sensitive taxonomic classification for metagenomics with Kaiju. Nat Commun.

[29] Mitra S, Drautz-Moses DI, Alhede M, Maw MT, Liu Y, Purbojati RW (2015). In silico analyses of metagenomes from human atherosclerotic plaque samples. Microbiome.

[30] Andrews S. FastQC: A Quality Control Tool for High Throughput Sequence Data. 2010. http://www.bioinformatics.babraham.ac.uk/projects/fastqc/

[31] Ewels P, Magnusson M, Lundin S, Käller M (2016). MultiQC: summarize analysis results for multiple tools and samples in a single report. Bioinformatics.

[32] Martin M (2011). Cutadapt removes adapter sequences from high-throughput sequencing reads. EMBnet J.

[33] Langmead B, Trapnell C, Pop M, Salzberg SL (2009). Ultrafast and memory-efficient alignment of short DNA sequences to the human genome. Genome Biol.

[34] Langmead B, Salzberg SL (2012). Fast gapped-read alignment with Bowtie 2. Nat Methods.

[35] Wood DE, Lu J, Langmead B (2019). Improved metagenomic analysis with Kraken 2. Genome Biol.

[36] Zhu Z, Ren J, Michail S, Sun F (2019). MicroPro: Using metagenomic unmapped reads to provide insights into human microbiota and disease associations. Genome Biol.

[37] Uritskiy GV, Diruggiero J, Taylor J (2018). MetaWRAP—a flexible pipeline for genome-resolved metagenomic data analysis. Microbiome.

[38] Clarke EL, Taylor LJ, Zhao C, Connell A, Lee JJ, Fett B (2019). Sunbeam: an extensible pipeline for analyzing metagenomic sequencing experiments. Microbiome.

[39] Ondov BD, Bergman NH, Phillippy AM (2011). Interactive metagenomic visualization in a Web browser. BMC Bioinform.

[40] Nurk S, Bankevich A, Antipov D, Gurevich A, Korobeynikov A, Lapidus A, et al. Assembling genomes and mini-metagenomes from highly chimeric reads. In: Lecture Notes in Computer Science (including subseries Lecture Notes in Artificial Intelligence and Lecture Notes in Bioinformatics). Springer, Berlin; 2013. p. 158–70.

[41] Merkel D (2014). Docker: lightweight Linux containers for consistent development and deployment. Linux J.

[42] Domenico P. Kraken2 & Kaiju pre-indexed databases. 2020 Sep 28 [cited 2020 Nov 17]; https://zenodo.org/record/4055180

[43] Domenico P. Test Dataset for HOME-BIO. 2020 Oct 1 [cited 2020 Nov 17]; https://zenodo.org/record/4061297

