# Supplementary material for: HOME-BIO (sHOtgun MEtagenomic analysis of BIOlogical entities): a specific and comprehensive pipeline for metagenomic shotgun sequencing data analysis
Source: BMC Bioinformatics. 2021 Jul 5;22(Suppl 7):106. doi: 10.1186/s12859-021-04004-y (PMC8256542; doi:10.1186/s12859-021-04004-y)
Supplement: Supplementary file 9 — Additional file 9. Tutorial and use examples of HOME-BIO. [file 12859_2021_4004_MOESM9_ESM.pdf]

HOME-BIO

sHOtgun MEtagenomic analysis of BIOlogical entities

Tutorial

INDEX

1. Introduction
2. Description
3. Installation
   - 3.1. Docker and Python installation
   - 3.2. Pipeline installation
4. Preparation
   - 4.1. Download the databases
   - 4.2. Prepare the config_file
5. How to run HOME-BIO
6. Output

1 – INTRODUCTION

This tutorial explains how to run an analysis using HOME-BIO with one test sample that it is possible to download on https://doi.org/10.5281/zenodo.4061297. The test sample is composed of two paired-end fastq files of human DNA, created *in silico*. Following, it is described how to download, install and run the pipeline.

2 - DESCRIPTION

HOME-BIO (sHOtgun MEtagenomic analysis of BIOlogical entities) is a modular and exhaustive pipeline for analysis of biological entity estimation, specifically designed for shotgun sequenced clinical samples. HOME-BIO analysis provides a comprehensive taxonomy classification by querying different databases and carries out the main steps of a metagenomics investigation.

HOME-BIO is a dockerized solution for metagenomics analysis. Inside a DOCKER image, UBUNTU with Python 3.8 and Anaconda 3 (V. 2020/02) have been installed. Inside the Anaconda environment, it has been installed common software in a metagenomic investigation (MultiQC, FASTQC, Cutadapt, Bowtie, Bowtie2, STAR, Kraken2, Bracken, SPAdes, Kaiju, and krona).

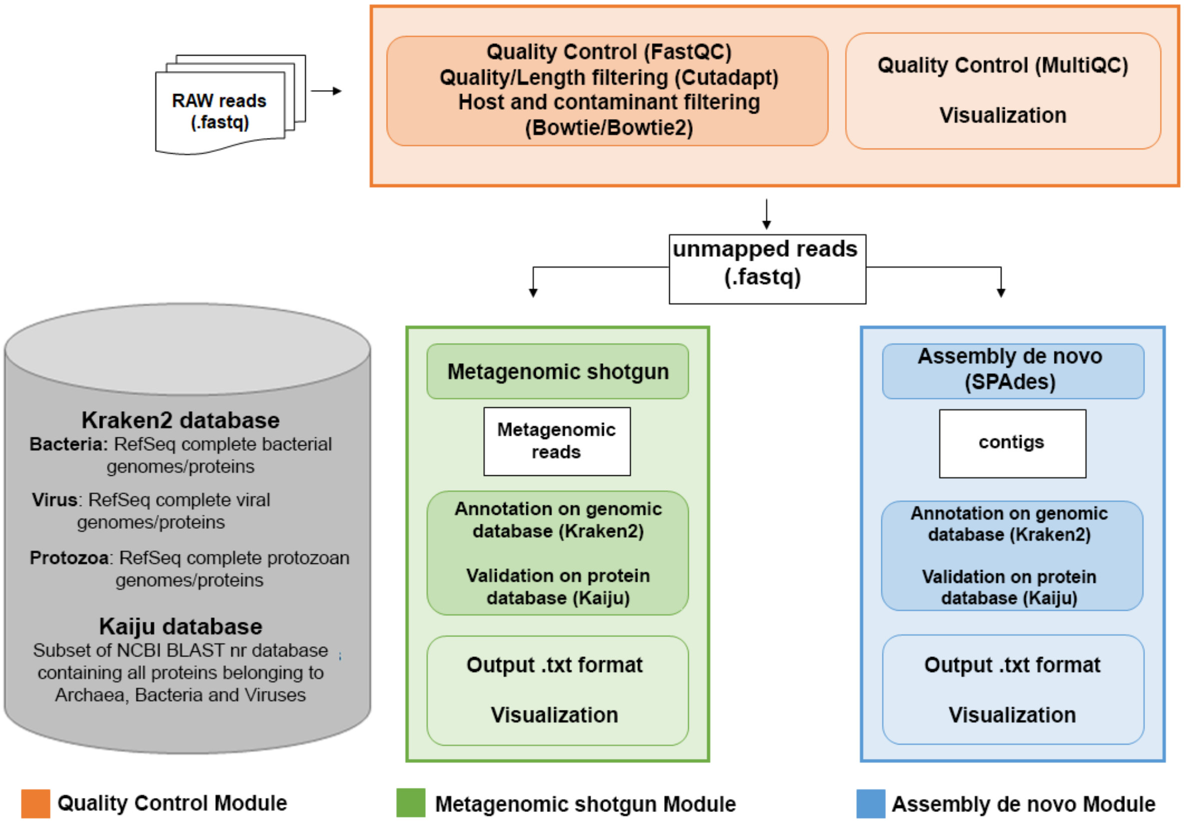

HOME-BIO workflow. The pipeline accepts NGS data as input and then proceeds to perform the analysis according to the different steps comprised in the three modules. The Quality Control module (in orange) performs QCs of the reads, low complexity reads removal, and host and contaminant sequencing read filtering (indicated in the dark orange rectangle), generating as output quality reports and graphical charts (indicated in the light orange rectangle). The sequence reads maintained after this step are processed using the Metagenomic shotgun module (in green) and/or the Assembly de novo module (in blue). In the first case, the reads are processed by Kraken2 and Kaiju (indicated in the dark green rectangle), producing as output text tables and charts. The Assembly de novo module processes the sequences, by assembling with SPAdes, and annotates with Kraken2 and Kaiju (dark blue rectangle), generating as output text tables and charts. For both modules, a custom database (indicates in the grey box) is used.

The pipeline takes in input fastq files.

- “Quality Control” module allows to perform sequence read quality checks and includes FASTQC and MultiQC, to perform the quality check and the summary of quality control, respectively, while the adapter trimming and removal of low-quality reads is performed by Cutadapt. If required, HOME-BIO performs a filtering step to remove host and contaminant sequence fragments by mapping each of them on the corresponding genome(s);

- “Metagenomic Shotgun” module performs taxonomic profiling, by classifying unmapped reads with Kraken2, and an additional protein-level classification with Kaiju;
- “Assembly de novo” module uses SPAdes (option --*meta*) for the assembly of unmapped reads; contigs sequence are generated and then classified as described before.

3 – INSTALLATION

3.1 – Docker and Python installation

To use HOME-BIO, it is mandatory to install and run DOCKER (https://hub.docker.com/) and install and use Python 3.

- At the following link (https://hub.docker.com/search?q=&type=edition&offering=community) you can find the correct version of DOCKER for your OS and all the info about how to install and run DOCKER.
- There are several options to install Python 3 depending on your OS:

Linux\Debian

```
sudo apt update
sudo apt-get install python3.8
```

Centos\RHEL\Fedora

```
yum update –y
yum install -y python3
```

Windows\MacOS

Just download and install it from https://www.python.org/downloads/windows/.

⚠ **For Windows users: during the installation, please, allow to add python in the path in order to run it directly by typing “`python`”.**

3.2 - Pipeline installation

Run DOCKER on your PC or server and pull our DOCKER image from https://hub.docker.com/r/biohaz/home_bio or just type in your console:

```
docker pull biohaz/home_bio:latest
```

Now, download the GitHub repository or, if you have git clone installed, type:

```
git clone https://github.com/carlferr/HOME-BIO.git
```

Nome	Tipo	Dimensione compr...	Protetto d...	Dimensione
config_file.txt	File TXT	1 KB	No	2 KB
Config_file_creator.py	File PY	1 KB	No	2 KB
Dockerfile	File	1 KB	No	1 KB
HOME_Bio.py	File PY	1 KB	No	3 KB
HOME_Bio_windows.py	File PY	1 KB	No	3 KB
LICENSE.txt	File TXT	3 KB	No	8 KB
README.md	File MD	3 KB	No	6 KB
Script.py	File PY	13 KB	No	217 KB

The figure above shows the content of the HOME-BIO-master.zip file downloaded from GitHub.

4 – PREPARATION

4.1 - Download the databases

To run HOME-BIO analysis, you should download indexed genome/protein reference databases for bacteria, protozoa, or viruses. We provide all of them on Zenodo (https://doi.org/10.5281/zenodo.4055180)

(`DB_KKRAKEN2_KAIJU.zip`). Download the zipped folder, and, before running the analysis, please unzip it on your machine.

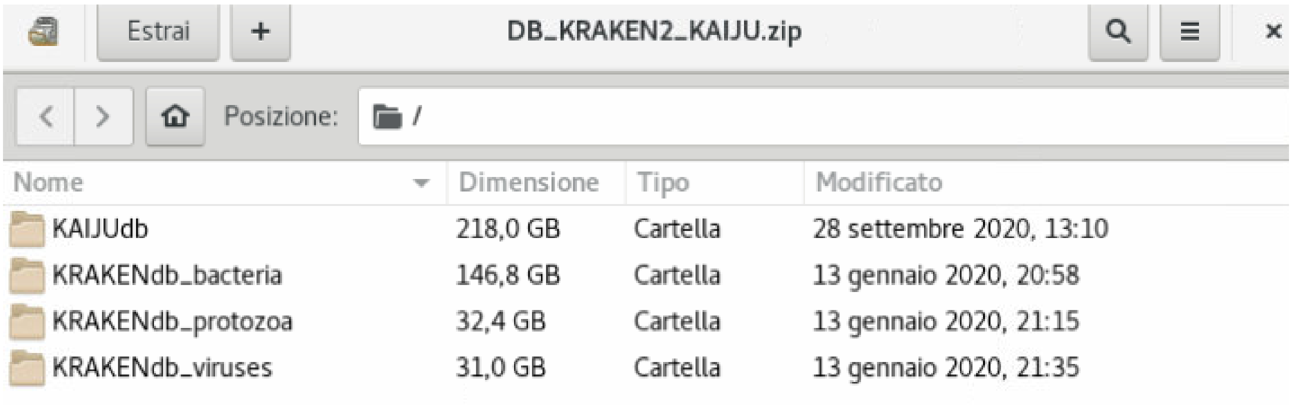

In the figure above, it is possible to see the content of the `DB_KRAKEN2_KAIJU.zip` file, available on Zenodo. Inside, there are three folders for Kraken2 and one folder for the Kaiju database.

⚠ **It is mandatory to have at least 400 GB of available space on the disk to extract the compressed folder. It is NOT mandatory to use our previously indexed databases, but the user will need to create his own databases and, then, index them with Kraken2 and Kaiju. See the instructions on Kraken2 (https://github.com/DerrickWood/kraken2/wiki/Manual) and Kaiju (https://github.com/bioinformatics-centre/kaiju) user manuals.**

4.2 - Prepare the Config file

Before running HOME-BIO, the users should manually change the "`config_file.txt`".

```
[DEFAULT]
quality control before trimming [yes/no] = no
quality control after trimming [yes/no] = no
number of threads [n] = 1
paired-end? [yes/no] = yes
filter out contaminant [yes/no] = no
adapter? = CTGTCTCTTATA
shotgun module [yes/no] = no
assembly denovo module [yes/no] = no
kraken2 confidence = 0.5
nucleic acid type [dna/rna] = DNA
k-mer [auto/21,33,55,77] = auto

[CUSTOM]
quality control before trimming [yes/no] = yes
quality control after trimming [yes/no] = yes
number of threads [n] = 16
path fastq = /root/Scrivania/HOME-BIO-master/fastq/
paired-end? [yes/no] = yes
path output = /home/genomi/output/
path host genome = /mnt/NFS_SHARE_2/Genomi/hg38_NCBI/NCBI/GRCh38/Sequence/Bowtie2Index/
filter out contaminant [yes/no] = no
path contaminant genome = /root/Scrivania/HOME-BIO-master/Genome/
adapter? = CTGTCTCTTATA
shotgun module [yes/no] = yes
assembly denovo module [yes/no] = yes
path kraken2 & kaiju databases = /root/Scaricati
kraken2 confidence = 0.5
nucleic acid type [dna/rna] = DNA
k-mer [auto/21,33,55,77] = auto
```

The figure above shows the content of the "`config_file.txt`" file.

This file is divided in two parts. The first `[DEFAULT]` indicates the default values for each parameter, while the second part `[CUSTOM]` should be modified by the user.

In particular, the user will manually modify, in the `[CUSTOM]` section, the correct options for the analysis, following the written examples. For some options an absolute path is mandatory, while for others it is required a "`yes`" or "`no`" selection. Only the "`Adapter`" option requires a sequence (in capital letters) to use as adapter during the trimming step; moreover, the adapter sequences may also contain any IUPAC wildcard character (such as N), as suggested in cutadapt user guide https://cutadapt.readthedocs.io/en/stable/guide.html. For the "`k-mers`" option of the Assembly de novo module, HOME-BIO is set to "`auto`". In this way, SPAdes will choose the best k-mer length depending on the reads length.

Only a comma-separated list of k-mer sizes can be used (all values must be odd, less than 128, and listed in ascending order, e.g. 21,33,55). For more details, see the SPADES manual (http://cab.spbu.ru/files/release3.13.0/manual.html). If the contaminant filtering is not requested, the "Path contaminant genome" is not used but it is still mandatory to write something (e.g., "/your_path/" or "none").

E.g. for "Number of threads [n] = " the user should insert, in the [CUSTOM] section, an integer number [n] indicating the max number of threads to be used (in this case 16 are used). For "path to host genome" is indicated the absolute path of Bowtie2 indexed host genome folder: "/root/Scrivania/HOME-BIO-master/Genome/".

The script Config_file_creator.py is able to re-generate the config file (in case it was lost or not usable anymore). The user will modify it as suggested before. Just type:

```
python Config_file_creator.py
```

It will create the "config_file.txt" or, if it is still present, rewrite it.

⚠ **Before running the analysis, remember to create the indexes for each genome (contaminant or reference) using Bowtie2 (**bowtie2-build [options]* <reference_in> <bt2_base>**); where** <reference_in> **is the genome sequences in fasta format and** <bt2_base> **is the basename of the index files to write (for detailed information see Bowtie2 manual** https://github.com/BenLangmead/bowtie2/blob/master/MAN%20UAL.markdown**).**

E.g.

```
bowtie2-build genome.fa host_genome_index
```

⚠ **If the user does not have a human reference sequence (here considered as host genome), he should follow the next step in order to download it from NCBI, decompress the genome sequence file (Homo sapiens - assembly GRCh38.p13), and, finally, create the proper index in the correct folder.**

E.g.

```
wget
https://ftp.ncbi.nlm.nih.gov/genomes/all/GCF/000/001/405/GCF_000001405.3
9_GRCh38.p13/GCF_000001405.39_GRCh38.p13_genomic.fna.gz

gunzip -f GCF_000001405.39_GRCh38.p13_genomic.fna.gz

mkdir ./Genome

bowtie2-build GCF_000001405.39_GRCh38.p13_genomic.fna
./Genome/host_genome_idx
```

In the config_file example above, options are set in order to:

- perform a quality check before and after the trimming
- run the analysis with 16 threads
- use the reads in a folder in paired-end mode (here, two paired-end fastq test files were used which can be downloaded on https://doi.org/10.5281/zenodo.4061297)
- use the human genome as host-genome (hg38 downloaded from NCBI and pre-indexed with Bowtie2)
- do not use a contaminant genome and do not perform the filtering
- use a specific adapter
- use the shotgun module and the assembly de novo module
- use our pre-indexed kaiju and kraken2 databases (downloaded and unzipped from Zenodo)
- use the default value of confidence for Kraken2 taxonomy annotation (0.5)
- set "DNA" as nucleic acid (since our test sample is DNA)
- do not use a custom set of kmers length.

If the user knows about a possible contaminant in the samples, he should indicate an absolute path of the pre-indexed genome with `path contaminant genome` option and he should use Bowtie2 in the same way the host genome was created (`bowtie2-build [options]* <reference_in> <bt2_base>`). We provide an example of a pre-indexed contaminant genome available on Zenodo (http://doi.org/10.5281/zenodo.4281135). Users should unzip the file and move the content in a folder (e.g. "`/root/Desktop/HOME-BIO-master/contaminant_genome/`"). Remember to compile the config file with this absolute path.

5 – HOW TO RUN HOME-BIO

HOME-BIO uses fastq (or fastq.gz) files as input. Remember to put all your input data in one unique folder (e.g., "Fastq_folder", "Data", or “Input").

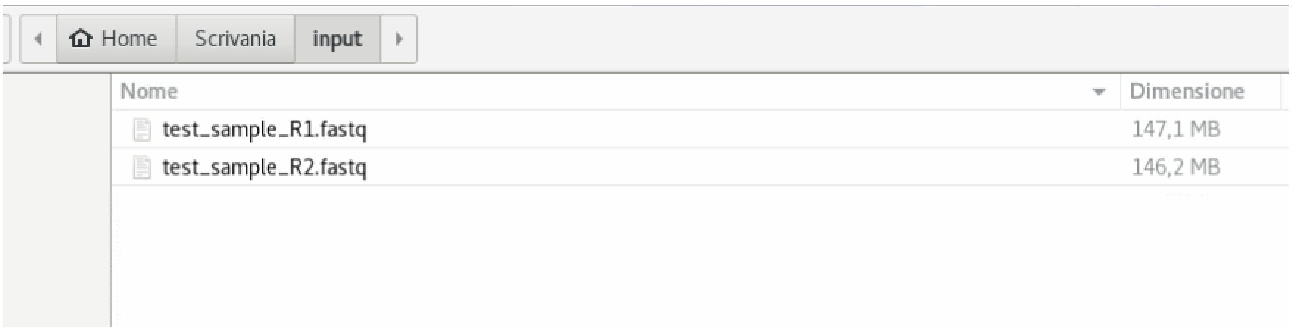

It is possible to test the pipeline using a testing dataset (two paired-end fastq files) freely available (https://doi.org/10.5281/zenodo.4061297) on Zenodo. The figure above shows the paired-end test sample in one unique folder.

Now, you can run HOME-BIO. Please, move in the HOME-BIO-master directory and just type in the console (for Linux\Debian\Centos\RHEL\Fedora):

```
python HOME_Bio.py -c config_file.txt
```

For Windows users, type

```
python HOME_Bio_windows.py -c config_file.txt
```

Running this command in your console, it will automatically determine: call of the Docker container, read of the paths from the `config_file.txt`, and launch of the analysis. During the analysis, several tools will be called and, after each one, a message indicating if the tool worked correctly is prompted. A log file will be generated in the output directory (“`log.txt`”) indicating, for each step, if some errors occurred.

6 - OUTPUT

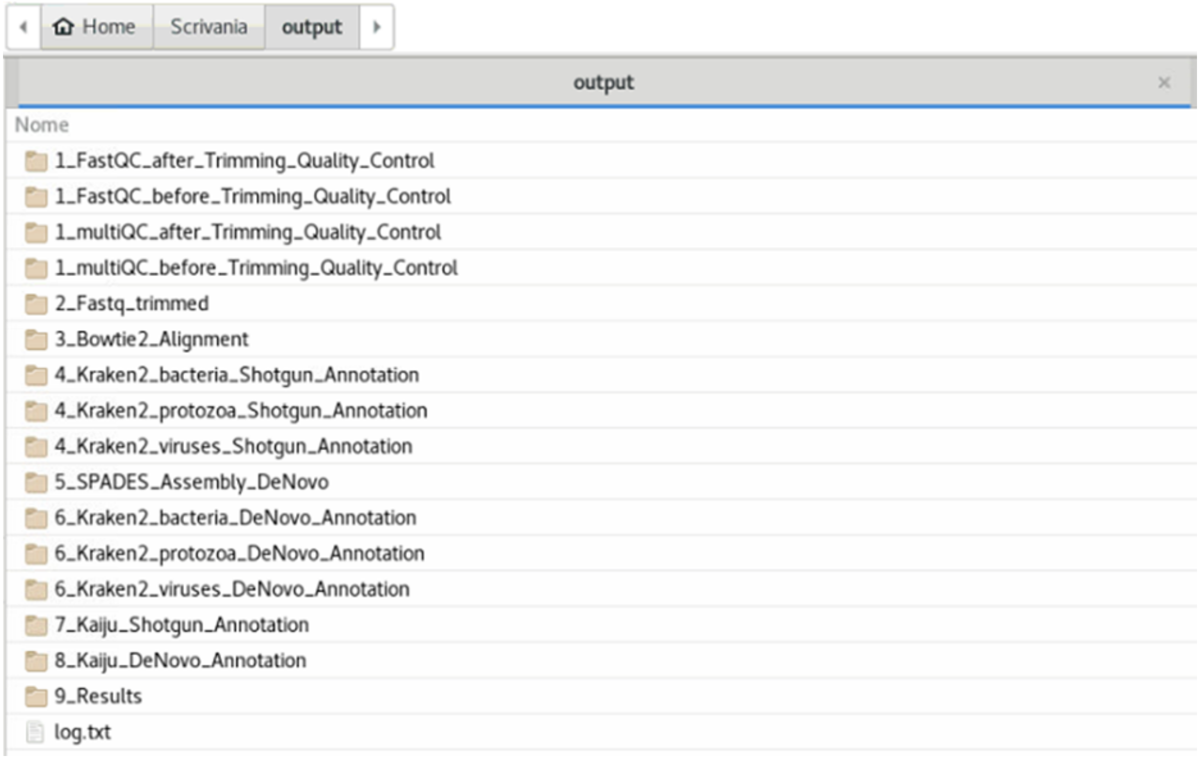

HOME-BIO modules (Shotgun metagenomic and Assembly de novo) will produce output files in tab-delimited (.txt) and graphic (.png) format. In the output folder (called "`output`" in this case), it is possible to find numbered folders for each tool used (e.g. "`3_Bowtie2_Alignment`" with all the files generated from the alignment step). In detail:

1. FastQC and MultiQC output files with quality control report of samples (in html and tab delimited-format), before and after adapter sequence removal. It is also provided a quality check summary of all the fastq samples analyzed in HOME-BIO;
2. Fastq files obtained after low quality read filtering and the adapter sequence removal procedures;
3. Alignment of output file obtained with the host and the contaminant filtering step. For each sample processed in HOME-BIO, metrics, alignment files (in sam format), fastq files with aligned fragment, and unmapped reads (used in next steps) are generated.
4. Metagenomic annotation output files from the Shotgun module. These folders contain classified and unclassified read sequences (in fastq format) obtained by querying HOME-BIO databases. Furthermore, there are also some intermediate files used to perform the taxonomy classification (see "`9_Results`" explanation).

5. Assembly de novo output files. For each sample analyzed, users can find metrics and parameters information of the de novo assembly step. In particular, `contig.fasta` and `scaffold.fasta` files contain all the sequences assembled with SPAdes.
6. Metagenomic annotation output files for the Assembly de novo module (as described before).
7. Protein-level annotation output files for the Shotgun module. This folder contains the summary files of the protein-based profiling reporting the detected entities and the related number (and percentage) of the reads mapping in HOME-BIO database; html interactive pie-chart, with all the entities found in the samples; tab-delimited output files with one line per reads processed indicating taxonomy classification results (name of the read, NCBI taxon IDs of identified taxa, the length or score of the best match used for the classification and the matching sequences).
8. Protein-level annotation output files for the Assembly de novo module (as described before).

HOME-BIO generates also a `log.txt` file containing all the messages prompted by the script during the analysis.

The "`9_Results`" folder is the final output, resulting from each module performed in HOME-BIO. In this tutorial, both modules were used:

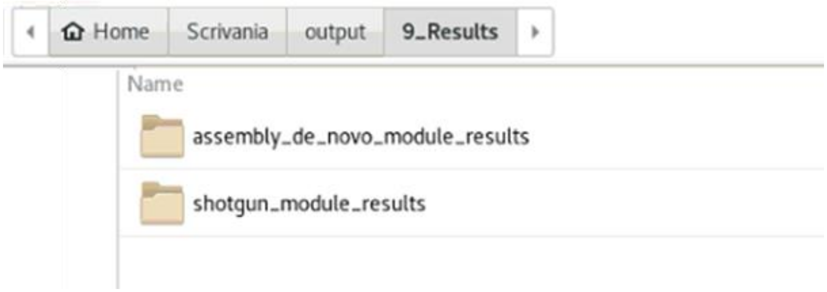

For each sample processed, it is possible to obtain the following resulting files. The figure below shows the content of the `assembly_de_novo_module_results` folder. The identical scheme is present in the `shotgun_module_results`.

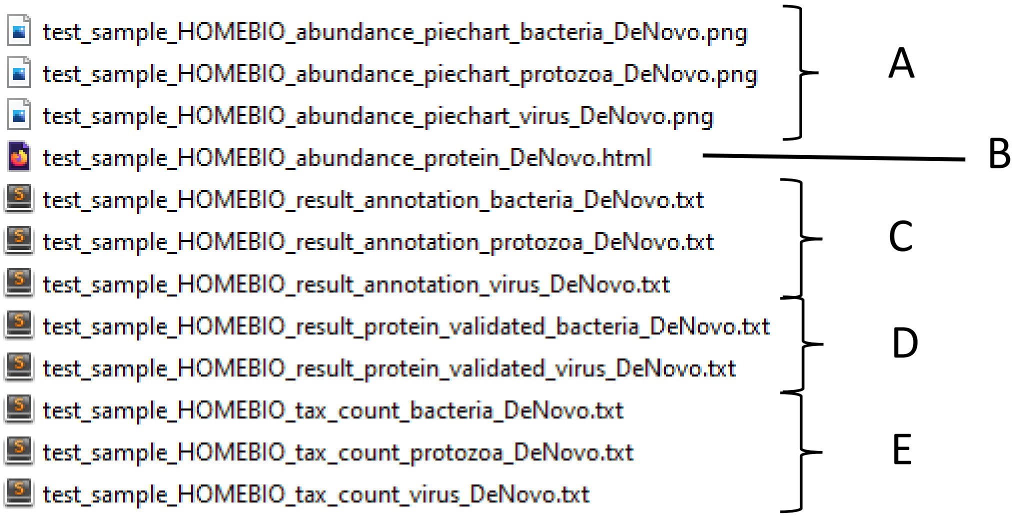

In detail:

A. `*_HOMEBIO_abundance_piechart*`: Pie charts with abundant estimation, reporting top abundant entities (bacteria, protozoa, and viruses) as shown in the following figure for bacteria annotation.

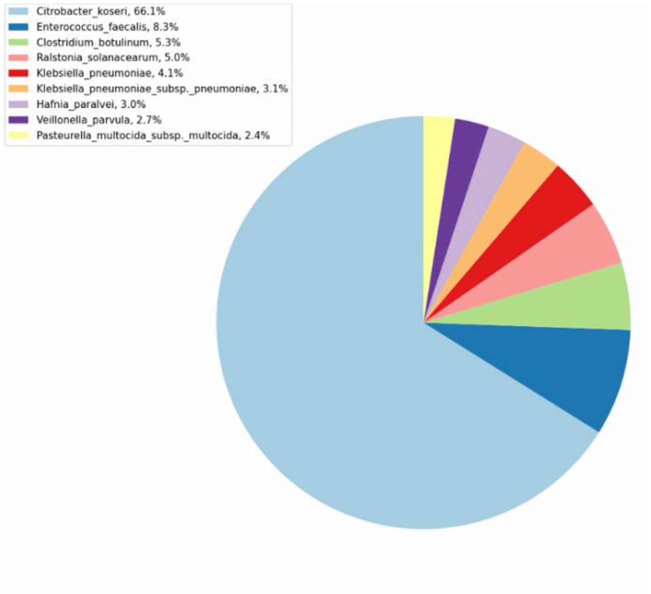

B. `*_HOMEBIO_abundance_protein*`: Multi-layered pie charts in .html format, reporting entities detected with a reference database of protein sequences from microbial and viral genomes. The interactive charts are self-contained and can be displayed with any modern web browser. Zooming on the interactive pie chart, the user can explore the taxonomic content of a sample;

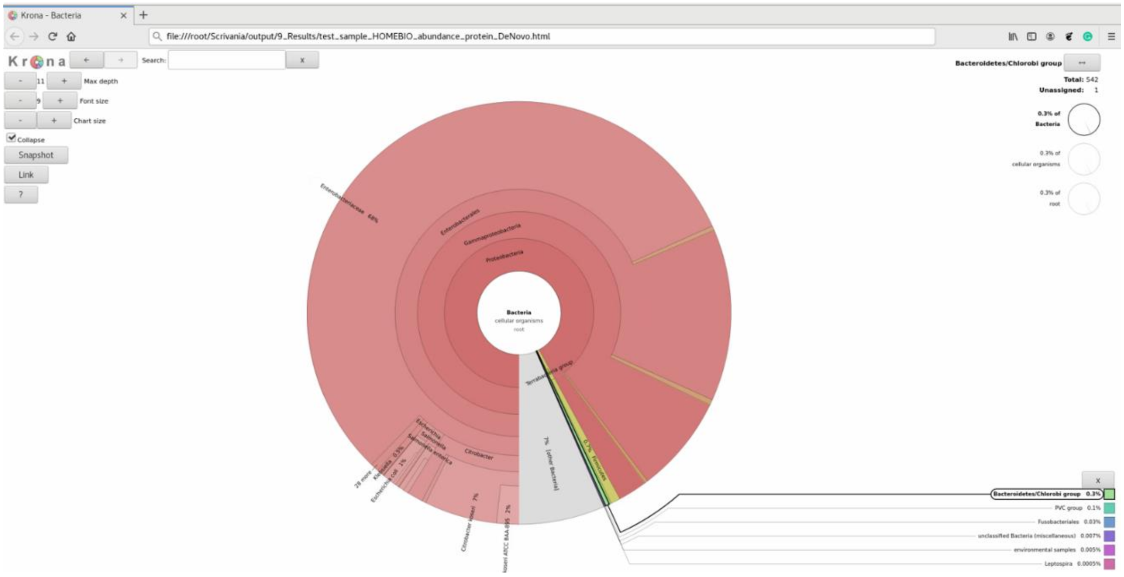

C. `*_HOMEBIO_results_annotation*`: these sample reports are in tab-delimited format with one line per taxon. Each one contains the information about the taxonomy classification of the samples (number and percentage of reads related to taxa in the reference database) as shown in the next example. The fields of the file, from left-to-right, are as follows:

1. Percentage of fragments covered by the clade rooted at this taxon.

2. Number of fragments covered by the clade rooted at this taxon.

3. Number of fragments assigned directly to this taxon.

4. A rank code, indicating (U)nclassified, (R)oot, (D)omain, (K)ingdom, (P)hylum, (C)lass, (O)rder, (F)amily, (G)enus, or (S)pecies.

5. NCBI taxonomic ID number.

6. Indented scientific name. The scientific names are indented using space, according to the tree structure specified by the taxonomy.

1	2	3	4	5	6
0.22	505	505	U	0	unclassified
99.78	233805	2159	D	2	Bacteria
97.44	228316	2001	P	1224	Proteobacteria
96.14	225272	2150	C	1236	Gammaproteobacteria
94.99	222569	18030	O	91347	Enterobacterales
86.41	202474	41447	F	543	Enterobacteriaceae
68.20	159802	154415	G	544	Citrobacter

D. `*_HOMEBIO_results_protein_validated*`: these sample reports have the same structure of the outputs mentioned above in the **C** section. In addition, they contain the 7th column reporting the protein validation information. If a given taxon finds a match both in the protein validation step with Kaiju annotation and in the Kraken2 taxonomy annotation with a different reference database, it will be "protein_validated"; as in example below for bacteria *Citrobacter koseri*.

1	2	3	4	5	6	7
4.65	51	51	U	0	unclassified	
95.35	1046	0	R	1	root	
95.35	1046	0	R1	131567	cellular organisms	
95.35	1046	0	D	2	Bacteria	
94.80	1040	21	P	1224	Proteobacteria	
92.89	1019	11	C	1236	Gammaproteobacteria	
91.80	1007	64	O	91347	Enterobacterales	
85.05	933	125	F	543	Enterobacteriaceae	
73.02	801	769	G	544	Citrobacter	
2.83	31	30	S	545	Citrobacter koseri	Protein_validated

E. `*HOMEBIO_tax_count*`: sample reports in tab-delimited format reporting number of genera and species (and related taxonomy) found in the samples.

73.02	801	769	G	544	Citrobacter
2.83	31	30	S	545	Citrobacter koseri
0.09	1	1	S1	290338	Citrobacter koseri ATCC BAA-895
0.09	1	0	G1	1344959	Citrobacter freundii complex
0.09	1	1	S	57706	Citrobacter braakii
0.36	4	3	G	570	Klebsiella
0.09	1	1	S	573	Klebsiella pneumoniae
0.27	3	2	G	561	Escherichia
0.09	1	1	S	562	Escherichia coli
0.27	3	0	G	204037	Dickeya
0.27	3	3	S	2037915	Dickeya sp. Secpp 1600
0.09	1	0	G	568	Hafnia
0.09	1	1	S	546367	Hafnia paralvei
0.09	1	0	G	745	Pasteurella
0.09	1	0	S	747	Pasteurella multocida
0.09	1	1	S1	44283	Pasteurella multocida subsp. multocida
0.27	3	0	G	1350	Enterococcus
0.27	3	3	S	1351	Enterococcus faecalis
0.18	2	0	G	1386	Bacillus
0.18	2	0	G1	86661	Bacillus cereus group
0.09	1	1	S	1396	Bacillus cereus
0.09	1	0	S	1428	Bacillus thuringiensis
0.09	1	0	S1	180877	Bacillus thuringiensis serovar pulsiensis
0.09	1	1	S2	527028	Bacillus thuringiensis serovar pulsiensis BGSC 4CC1
0.09	1	0	G	216851	Faecalibacterium
0.09	1	1	S	853	Faecalibacterium prausnitzii

Tot G=11 Tot S=15

For information contact: ggiurato@unisa.it, aweisz@unisa.it
